# The utility of mHealth applications in pressure ulcer management: A scoping review

**DOI:** 10.1177/20552076251386656

**Published:** 2025-11-25

**Authors:** Shreenidhi Jogi, Elsa Sanatombi Devi, Vishal Shanbhag, Ajitha Shenoy K B

**Affiliations:** 1 Department of Medical-Surgical Nursing, 76804 Manipal College of Nursing, Manipal Academy of Higher Education, Manipal, Karnataka, India; 2 Department of Critical Care Medicine, 29224 Kasturba Medical College, Manipal Academy of Higher Education, Manipal, Karnataka, India; 3 Department of Information and Communication Technology, 125853 Manipal Institute of Technology, Manipal Academy of Higher Education, Manipal, Karnataka, India

**Keywords:** mHealth, pressure ulcer, mobile applications, wound care, prevention, digital health, scoping review

## Abstract

**Background:**

Pressure ulcers are a persistent challenge in clinical and long-term care, often requiring timely detection, continuous monitoring, and consistent prevention strategies. As mobile health (mHealth) applications become more integrated into healthcare, there is growing interest in their potential to support pressure ulcer care, especially in settings with limited access to specialist expertise.

**Methods:**

A scoping review was conducted following the Arksey and O’Malley framework and PRISMA-ScR guidelines. Five databases (PubMed, Scopus, Web of Science, CINAHL, and EMBASE) were searched up to April 2025. Eligible studies included interventional and pilot technical validation trials evaluating mHealth applications used by healthcare professionals or caregivers for pressure ulcer care. Data were charted and summarized narratively with a focus on assessment, monitoring, and prevention features.

**Results:**

Four studies were included in the review. The mHealth interventions varied in delivery and design, supporting clinical assessment including educational modules, staging tools, artificial intelligence (AI)-assisted wound analysis, and caregiver-focused applications. Outcomes reported across studies included improved knowledge among nurses and caregivers, greater caregiver self-efficacy, enhanced documentation accuracy, and significant wound size reduction. Despite heterogeneity in study designs, populations, and outcomes, all interventions demonstrated positive impacts on pressure ulcer management.

**Conclusion:**

mHealth applications show promising potential to enhance pressure ulcer assessment, monitoring, and prevention. These tools provide accessible support for clinicians and caregivers. However, the evidence base remains small, heterogeneous, and largely from pilot or quasi-experimental studies, with some apps still in early validation stages. Larger, long-term evaluations are needed to establish clinical effectiveness, cost-effectiveness, and integration into routine workflows.

## Introduction

Pressure ulcers are recognized as one of the most expensive and physically exhausting medical conditions in the twentieth century.^1^ A pressure ulcer injury is a confined injury to the skin, typically occurring over a bony prominence because of continuous pressure that is unrelieved. Pressure ulcers are also known as decubitus ulcers or pressure injuries.^2^ These ulcers are caused by intrinsic factors such as reduced mobility, underlying health conditions, aging skin and inadequate nutrition and extrinsic factors like pressure, shear, friction, and moisture.^3^ Globally, there were 3,170,796 new cases of pressure ulcers in 2019, with 55% of them occurring in men and 45% in women, and the majority occurring in people over 75 years old.^4^ A meta-analysis reports that pressure ulcers have a global prevalence of 12.8%, with hospital-acquired pressure injuries (HAPIs) accounting for 8.4% of cases.^5^ On average, stage II pressure ulcers require approximately 23 days for complete re-epithelialization. However, smaller ulcers tend to heal approximately 12 days faster compared to larger ones with a surface area of 3.1 cm or greater.^6^ Common strategies for preventing pressure ulcers include the use of appropriate support surfaces, regular patient repositioning, optimized nutrition, and proper skin care. Additionally, conducting risk assessments and maintaining standardized documentation are essential for early detection and effective management of pressure ulcers.^7^ The detailed documentation of pressure ulcer characteristics, progression, and care interventions is vital for effective treatment planning and progress evaluation of pressure ulcers.^3,8^

Early detection of pressure ulcers and effective treatment based on accurate diagnostic results are crucial for preventing ulcer progression and preventing the long-term burden of pressure ulcers.^9^ Regular monitoring of the pressure ulcer's progression by physicians and healthcare professionals is essential in pressure ulcer management. However, in-person evaluation by specialists is not always feasible, particularly for patients lacking access to specialized transportation, those without family support, or those living in remote areas.^10^ Managing pressure injuries in the elderly is becoming increasingly important in an aging population. Proper classification of pressure injury stages is crucial for effective wound care planning. However, the specialized expertise needed for accurate staging is often unavailable in residential care home settings.^8^

Telemedicine, telehealth, and electronic health (eHealth) refer to the remote application of information and communication technologies to facilitate healthcare delivery. These technologies assist in the diagnosis, treatment, monitoring, and prevention of diseases or injuries. These technologies also help in health education for caregivers, which enhances the wellbeing of individuals and communities.^11^ Smartphones have become more accessible for people. The overall usage of smartphones for various purposes has increased worldwide. This has also contributed for the use of mobile applications for managing health conditions, disease and injuries. At the same time, access to these technologies is not universal, and certain populations such as older adults, individuals in low-resource settings, or those with limited technological familiarity may encounter barriers to engaging with smartphone-based health tools. The World Health Organization (WHO) defines mobile health (mHealth) as the practice of medicine and public health supported by mobile devices, such as smartphones and other wireless technologies.^12^ The role of mHealth apps in healthcare is significantly growing.^10,13–15^ mHealth apps enable physicians and healthcare personnel to remotely assess, monitor, and analyse the progress of the pressure ulcers. This indeed improves pressure ulcer evaluation in clinical, hospital, and home-based settings. It is easy to update the information and resources in smartphones with the help of an internet connection as compared to the traditional paper-based methods. mHealth apps also enable users to develop skills, gain confidence, and quickly adapt to the technology.^10,16–18^ mHealth apps can provide personalized self-management support for individuals with chronic health conditions by offering tailored education, reminders, and monitoring tools that assist patients in preventing pressure injuries.^19^

Several feasibility studies have looked at the use of mHealth tools for wound monitoring and documentation, and most have shown that these applications are not only practical but also reasonably reliable in real-world care. The imitoMeasure app, for example, demonstrated excellent validity and reproducibility when compared with traditional measurement methods.^20^ Similarly, Amann et al.^19^ reported that a self-management app designed for people with spinal cord injury was both feasible and acceptable for supporting pressure injury prevention. McKeown et al.^16^ likewise reported on the usability of mobile wound imaging tools in clinical practice. Moving beyond feasibility, implementation studies point to barriers that may slow or limit adoption. These include low digital literacy among some users, difficulties in linking apps with existing workflows, and concerns about user trust, all of which shape the adoption and long-term sustainability of these technologies.^17,18^ Overall, this suggests that mHealth applications have considerable potential but also face contextual challenges that must be resolved before they can be widely adopted in pressure ulcer care. Other research into electronic decision-support tools shows promising outcomes in improving clinician adherence to prevention guidelines and reducing care costs, although prevalence and incidence outcomes were inconsistent.^21^ The wider digital health literature further echoes these issues, often pointing to challenges with building user trust, ensuring smooth integration into existing clinical systems, and achieving sustainable deployment over time.^22^ For this reason, developing confidence in mHealth solutions among both patients and healthcare professionals is essential if they are to be successfully scaled and embedded into care.^19^

Koepp et al.^10^ conducted a systematic review and a survey in the app stores to evaluate the mHealth apps designed to identify, assess, treat, and prevent pressure ulcer injuries in adults. The review highlighted that all studies focused on the initial phases of app development and process of developing those apps and their validation. Therefore, the use of mobile apps for identifying, assessing, treating, and preventing pressure ulcers in adults remains limited. However, the reviewed studies offered valuable insights for future research. Additionally, mHealth apps should be evaluated in real-world healthcare settings across diverse populations, taking different ethnicities into account to ensure their relevance and usability for patients, caregivers, healthcare professionals, and students. Although the widespread availability of smartphones has created new opportunities for mHealth solutions, accessibility remains uneven. A recent study reported that major app stores are not organized in a way that enables patients, caregivers, or even clinicians to readily identify high-quality medical applications, thereby creating a barrier to adoption and effective use.^23^

More recent reviews have broadened the scope of digital health research in wound care beyond the work of Koepp et al.^10^ For example, Dege et al.^14^ systematically examined publicly available patient-centred chronic wound apps, with particular attention to their features, usability, and app-store quality, rather than their clinical effectiveness. Griffa et al.^13^ provided a narrative review of artificial intelligence (AI)-enabled ulcer segmentation applications, highlighting their technical potential while also noting persistent gaps in the transparency of underlying datasets and algorithms. Similarly, Gagnon et al.^15^ surveyed wound care applications designed for nurses, underscoring aspects such as availability, self-support functions, and evaluation criteria relevant for integration into practice.

Developing mHealth apps specifically designed to cater for pressure ulcer management offer significant benefits to the patients, physicians, healthcare professionals, and caregivers. These apps allow caregivers to confidently care for pressure ulcers in a home setting, while healthcare professionals can remotely monitor the progress through the app. Such innovative apps can improve care and help prevent, assess, monitor, and manage pressure ulcers more effectively. Through the literature review, it was identified that various studies and reviews have only focused on development and validation of apps for pressure ulcer care. Previous reviews have highlighted the growing use of mobile and digital technologies in wound care, with most emphasizing feasibility, usability, or app development rather than clinical effectiveness.^10,13,14^ Recent studies have drawn attention to the perspectives of nurses and caregivers in the adoption of such technologies.^15^

Despite these contributions, there remains a notable gap in the literature regarding how mHealth applications support the core domains of pressure ulcer care, including assessment, monitoring, prevention, and management, across diverse care contexts. To address this, the present scoping review synthesizes interventional evidence specific to pressure ulcers and maps how mHealth solutions have been implemented across various settings and populations. The objective of this review is to examine how these tools are applied to support assessment, monitoring, prevention, and management strategies across various care settings and populations. Additionally, this review identifies existing knowledge gaps and highlights priority areas for future research and innovation to enhance the role of digital health in pressure ulcer prevention and care.

## Materials & methods

This review was conducted following the Arksey and O’Malley framework,^24^ with refinements and reported using the PRISMA-ScR guidelines.^25^

### Identifying the research question

This review aimed to examine how mHealth applications have been applied in the care of pressure ulcers and to map the variety of outcomes reported in existing studies. Rather than focusing solely on effectiveness, the goal was to provide a broad overview of current research activity, identify shared features among interventions, and highlight areas where more evidence is needed to guide future development and implementation.

### Eligibility criteria

This review included randomized controlled trials, nonrandomized controlled trials, and pilot technical validation trials that evaluated the use of mobile applications in the care and management of pressure ulcers. Studies such as systematic reviews, qualitative research, mixed-methods studies, conference proceedings, and descriptive studies were excluded. Eligible studies specifically focused on patients with pressure ulcers and involved healthcare providers or caregivers utilizing mobile health applications to support the care and management of pressure ulcers.

### Information sources

A comprehensive search was conducted across several literature databases, including CINAHL (via EBSCOhost), MEDLINE (via PubMed), Scopus, Web of Science, and EMBASE. Studies published up to April 2025 were considered, with no restrictions on the starting publication date. The database search was conducted in April 2025. Only English-language studies available in free full-text were includedto ensure complete access to study data for extraction and synthesis.

### Search strategy

The search strategy combined Medical Subject Headings (MeSH) with relevant keywords to ensure a broad and inclusive search. The terms used included “pressure ulcer,” “pressure injury,” “bedsores,” “decubitus ulcer” along with terms related to mobile health, including “mobile health,” “mHealth,” and “mobile applications.” Additional keywords like “care,” “assessment,” “prevention,” and “management” were also incorporated to capture studies focused on different aspects of pressure ulcer care.

### Study selection

The study selection followed the PRISMA-ScR guidelines,^25^ to ensure a transparent and systematic process. Comprehensive searches were conducted across five major databases, and all retrieved records were imported into Rayyan.^26^ Duplicate entries were removed, after which two reviewers independently screened the titles and abstracts using predefined inclusion criteria. Studies were considered eligible if they focused on mHealth applications specifically developed for the assessment, monitoring, or prevention of pressure ulcers and reported outcomes such as usability, clinical effectiveness, or user engagement. Full texts were then reviewed for articles that passed the initial screening, and any disagreements regarding study eligibility were resolved through discussion. Out of 243 records initially retrieved, nine articles were selected for full-text screening, and four studies met all criteria and were included in the final synthesis.

### Data charting

Data charting was carried out using a structured extraction framework aligned with the objectives of this review. Key information was extracted from each including study, including author and year, country of origin, study design, population and setting, sample size, intervention details, comparator, duration of the intervention, components of the intervention, and key findings. In addition, technical and delivery features of each mHealth application such as platform type, level of interactivity, and intended user group were charted separately to provide a more comprehensive overview. This process enabled systematic comparison across studies, despite heterogeneity in design and focus. Data charting was performed by one reviewer and verified by a second to ensure accuracy and completeness. The charted data formed the basis for the thematic synthesis presented in the results. The themes assessment, monitoring, and prevention, with the help of mHealth apps were identified deductively, based on the predefined objectives of the review and aligned with the primary functionalities of the included mHealth applications.

## Results

### Identification of studies

**Figure 1.**
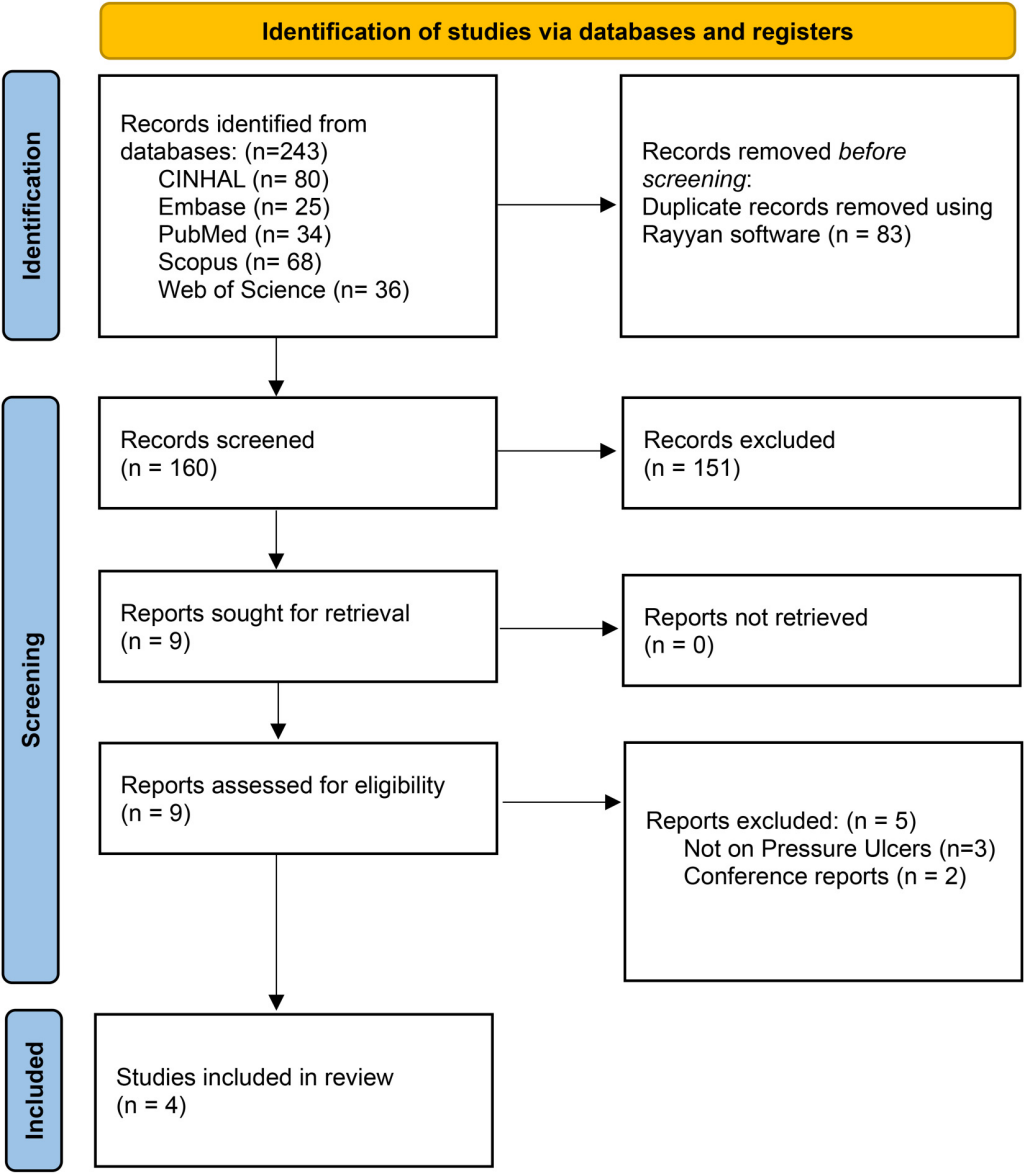
PRISMA-ScR flow diagram for the detailed procedures of study selection.

A total of 243 records were identified through comprehensive searches conducted across five electronic databases including PubMed, Scopus, Web of Science, EMBASE, and CINAHL. No restrictions were applied regarding publication year to ensure broad coverage, although the search was limited to articles published in English. After the removal of duplicates, 95 records were included for screening. Two reviewers independently screened the titles and abstracts to assess their relevance based on predefined inclusion criteria. Eligible study designs included randomized controlled trials, quasi-experimental studies (interventional studies without random assignment of participants to groups), and pilot studies. Following title and abstract screening, nine studies were selected for full-text review. Each full text was assessed independently by two reviewers, with discrepancies resolved through discussion and, when necessary, by involving a third reviewer. In total, four studies met all inclusion criteria and were included in the final synthesis. These studies represented a range of methodological designs, including two quasi-experimental studies, one pilot technical validation trial, and one fully powered randomized controlled trial. Each evaluated a distinct mHealth application aimed at pressure ulcer management, targeting different user groups such as nurses, caregivers, and patients across hospital and home-based care settings. The selection process is depicted in the PRISMA-ScR diagram^27^ presented in Figure 1.

### Characteristics of selected studies

The four studies included in this review were published between 2022 and 2024 and were conducted in Taiwan, Hong Kong, Australia, and Cyprus. The methodological designs comprised two quasi-experimental studies one one pilot technical validation study, and one randomized controlled trial. The target populations varied across studies and included nurses working in acute care settings, hospital in-patients with existing pressure ulcers, and caregivers providing home-based care and. Sample sizes ranged from 46 to 78 participants, with intervention and control groups appropriately balanced. Each study evaluated a distinct mHealth application developed for pressure ulcer care, encompassing a range of functions such as AI-assisted wound documentation, educational interventions, risk assessment tools, and image-based detection systems. Reported outcomes reflected positive impacts such as improvements in nurses knowledge and confidence in pressure ulcer staging,^28^ the demonstrated feasibility of real-time, AI-based pressure injury staging classification,^8^ enhanced wound documentation quality and faster healing rates,^29^ and increased caregiver knowledge, self-efficacy, and app acceptance over time.^30^ A detailed summary of the study characteristics is presented in Table 1.

**Table 1.** Characteristics of included studies.

Author (year)	Country	Study design	Population and setting	Sample size
Chuang et al. (2022)^28^	Taiwan	Randomized controlled trial	Nurses in acute care settings	IG: 86 / CG: 78
Lau et al. (2022)^8^	Hong Kong	Pilot technical validation study	Publicly available image datasets	144 (Images)
Barakat-Johnson et al. (2022)^29^	Australia	Quasi-experimental	Patients with pressure ulcers in hospital and community	IG: 124 / CG: 166
Polychronis et al. (2024)^30^	Cyprus	Quasi-experimental	Home caregivers for PU patients	IG: 23 / CG: 22

CG: control group; IG: intervention group.

The included studies varied in how they described the underlying conditions of participants. Barakat-Johnson et al.^29^ focused on hospital in-patients and outpatients with existing pressure ulcers and other types of wounds. Lau et al.^8^ did not study clinical population directly but instead used publicly available image datasets to train and validate their AI model. Polychronis et al.^30^ evaluated the use of a caregiver-focused app in home settings, although comorbidities and specific risk factors were not consistently reported. In contrast, Chuang et al.^28^ targeted nurses in acute care settings and did not provide patient-level clinical details.

### Synthesis of results

Given the heterogeneity in study designs, target populations, and outcome measures, the findings were summarized narratively within this scoping review. The four included studies collectively evaluated the effectiveness of distinct mHealth applications aimed at supporting pressure ulcer prevention, assessment, or management across diverse care settings and user populations. Despite differences in study design and intervention components, all studies reported positive outcomes related to either clinical effectiveness, user engagement, knowledge improvement, or documentation practices. The mHealth applications were delivered via smartphones or tablets and were designed for independent use by nurses, caregivers, or patients. Intervention durations ranged from 1 to 4 months, with usage frequencies varying from daily to scheduled intervals. While the pressure injury E-book app was primarily educational, the other three incorporated interactive elements such as AI-based wound measurement, image-based detection, risk scoring, and caregiver prompts. Notably, connectivity requirements varied among the apps. The AI-staging app was designed to function offline, whereas the Tissue Analytics app was cloud-based and required an internet connection. A detailed overview of the interventions is presented in Table 2, and Table 3 expands on their technical and delivery features.

**Table 2.** Characteristics of intervention in the included studies.

Author (year)	Intervention	Comparator	Duration of intervention	Components of the intervention
Chuang et al. (2022)^28^	Pressure injury E-book app	Standard education (no app)	One month	Educational modules, quizzes, PU staging content
Lau et al. (2022)^8^	Custom PU detection app	Not available	One-time per assessment	AI-based image analysis for PU staging, wound photo capture
Barakat-Johnson et al. (2022)^29^	Tissue analytics app	Standard wound care (no app)	4 months	AI-based wound measurement, image capture, clinical documentation features
Polychronis et al. (2024)^30^	PressureUlcerAdvisor app	Informational booklet (no app)	4 months	Braden scale risk assessment, educational content, repositioning reminders

AI: artificial intelligence.

**Table 3.** Technical and delivery features of the included mHealth applications.

Author (year)	App name/intervention	Delivery platform	Connectivity	User type	Interactivity level
Chuang et al. (2022)^28^	Pressure injury E-book app	Smartphone/tablet	Not specified	Nurses	Moderately interactive
Lau et al. (2022)^8^	Custom PU detection app	Smartphone (Android)	Offline	Caregivers	Minimally interactive
Barakat-Johnson et al. (2022)^29^	Tissue analytics app	Smartphone/tablet, web portal	Requires internet (cloud connected)	Physicians, nurses	Highly interactive
Polychronis et al. (2024)^30^	PressureUlcerAdvisor app	Smartphone	Not specified	Informal caregivers	Moderately interactive

### Assessment capabilities of mHealth applications

The included studies approached pressure ulcer assessment through different mechanisms, including clinician education, risk scoring, and AI-based image analysis.The pressure injury E-book app significantly improved nurses knowledge in pressure ulcer staging and assessment, with postintervention knowledge test scores showing a statistically significant increase (p < 0.000).^28^ Another study targeted prevention in the community by providing informal caregivers with PressureUlcerAdvisor app that incorporated the Braden scale, a validated risk assessment tool that evaluates a patient's likelihood of developing pressure ulcers based on factors such as sensory perception, moisture, activity, mobility, nutrition, and friction/shear,^31^ for risk assessment, alongside educational content, and repositioning alerts.^30^ The remaining two studies focused on the assessment of wounds using AI. One was a pilot technical validation of a custom-built app that used a deep-learning model to perform real-time staging classification of pressure ulcers from an image.^8^ The other evaluated a cloud-based Tissue Analytics app in clinical practice that provided AI-assisted wound measurement, analysis, and documentation support, which was used by both nurses and physicians.^29^

### Pressure ulcer monitoring and documentation via mHealth

Two studies reported on wound monitoring using mobile applications.^29,30^ The Tissue Analytics app, which utilized AI for wound image analysis, and found significant improvements in wound documentation. The completeness of wound records including documentation of all required parameters such as wound size, depth, exudate, and tissue type increased from 24% in the control group to 93.5% in the intervention group (p < 0.001). Additionally, for patients with pressure ulcers in the intervention group, 44 of 58 wounds (76%) improved, with an average size reduction of 41.99%. A direct comparison of healing rates with the control group was not performed.^29^ Another study addressed preventative monitoring by providing caregivers with an app featuring a risk assessment tool and repositioning alerts, but it did not include features for tracking existing wounds.^30^

### Enhancing preventive practices

Two studies implemented preventive strategies using mobile apps.^28,30^ The PressureUlcerAdvisor app provided education, repositioning alerts, and a risk assessment tool. Caregivers using the app showed a statistically significant improvement in knowledge (p = 0.040), increased self-efficacy (p = 0.049), and greater acceptance of the app over time (p = 0.010). Additionally, no significant difference was found in the incidence of new pressure ulcers, while the control group saw an increase in nonblanchable erythema.^30^ The E-book app indirectly supported prevention by improving nurses confidence in performing timely repositioning and skin care, although no direct ulcer incidence outcomes were measured.^28^ Across all two studies, mHealth tools were found to play a supportive role in reinforcing best practices and enabling both formal and informal caregivers to implement prevention strategies more reliably. A summary of key findings from all four studies is presented in Table 4.

**Table 4.** Key findings of included studies.

Author (year)	Intervention	Key findings
Chuang et al. (2022)^28^	Pressure injury E-book app	Significant improvement in nurses knowledge on PU staging, attitude, confidence, and management as assessed by a structured knowledge test questionnaire called Knowledge of Pressure Injury Scale (KPIS) (p < 0.000)
Lau et al. (2022)^8^	Custom PU detection app	Demonstrated the technical feasibility of a real-time, AI-based staging app, reporting an overall accuracy of 63.2% on a validation image set
Barakat-Johnson et al. (2022)^29^	Tissue analytics app	Significant improvement in wound documentation (from 24.0% to 93.5%, p < 0.001); reported a 41.99% average wound size reduction for the pressure ulcer
Polychronis et al. (2024)^30^	PressureUlcerAdvisor app	Increased caregiver knowledge (p = 0.040), self-efficacy (p = 0.049), and app acceptance (p = 0.010); no significant difference was found in the incidence of new pressure ulcers

AI: artificial intelligence.

As summarized in Table 4, the included interventions demonstrated a range of positive impacts across different dimensions of pressure ulcer care. The pressure injury E-book app improved nurses knowledge and confidence in staging and management.^28^ The custom detection app demonstrated the feasibility of real-time pressure injury staging classification.^8^ The Tissue Analytics app enhanced wound documentation and supported positive healing outcomes, evidenced by significant wound size reduction.^29^ The PressureUlcerAdvisor app strengthened caregiver knowledge, self-efficacy, and acceptance of digital tools.^30^ Collectively, these findings suggest that mHealth applications can support knowledge acquisition, improve clinical documentation, enhance early detection, and empower caregivers in preventive and management practices.

## Discussion

This scoping review aimed to explore the role of mHealth applications in supporting the care of individuals with pressure ulcers, with particular emphasis on their use in assessment, monitoring, prevention, and overall pressure ulcer management. The synthesis of four interventional studies points to a growing momentum in the use of digital health solutions as a complement to conventional care practices. Taken together, the findings indicate that mHealth tools have the potential to streamline clinical workflows, build caregiver knowledge and confidence, and support more timely and consistent wound care. These findings align with feasibility and implementation studies in digital wound care, which emphasize both the potential of mobile technologies and the challenges of integration into routine practice. By situating interventional evidence within this broader context, the review highlights the promise of mHealth in addressing persistent gaps in pressure ulcer management, while also identifying critical considerations for future research and implementation.

### mHealth in pressure ulcer assessment

Accurate and timely assessment is the foundation of effective pressure ulcer management. Yet, in many settings especially where access to specialist expertise is limited, this can be a real challenge. Traditionally, assessment relies heavily on clinical judgment and experience, which may not always be available, particularly for nonspecialist staff or caregivers. This is where mHealth tools are beginning to make a meaningful difference. The pressure injury E-book app was introduced to help nurses better understand ulcer staging and care. The app not only improved knowledge but also gave nurses the confidence to carry out assessments more consistently, reinforcing the value of accessible, on-the-go learning in clinical practice.^28^ Likewise, a technical validation of a custom-built app using an AI model to perform real-time staging classification of pressure ulcers from an image reported its own performance metrics, including an overall accuracy of 63.2% on a validation image set, along with variable sensitivity and high specificity. As an early-stage technical validation, it demonstrated the feasibility of the concept but requires further clinical testing to establish its utility.^8^ These findings align with feasibility studies that have validated wound measurement apps like *imitoMeasure*, which showed strong accuracy and reproducibility compared with traditional methods.^20^ However, broader implementation research has highlighted challenges such as the need for adequate training and the integration of these tools into existing documentation workflows.^19,32^ Collectively, this evidence suggests that while digital solutions have the potential to enhance assessment accuracy, their long-term impact will depend on how effectively they are embedded within routine clinical practice. These mHealth apps can be especially helpful in home or community settings, where delays in identifying skin breakdown can lead to serious complications. By putting practical tools into the hands of those closest to the patient, mHealth is helping to bridge critical gaps in care and enabling earlier, more accurate interventions where they matter most. These findings underscore the value of mHealth tools in strengthening early detection and promoting consistent pressure ulcer assessment, especially in low-resource and community-based settings.

### mHealth in monitoring pressure ulcer progression

Monitoring the healing of pressure ulcers is a continuous process that requires consistent documentation and careful observation for subtle changes over time. This can be difficult to maintain especially when care is fragmented across multiple providers or delivered in home settings where resources are limited. mHealth tools are beginning to close this gap by offering structured, easy-to-use platforms that support continuous pressure ulcer monitoring. The Tissue Analytics app, an AI-powered tool that captures wound images and analyses them using structured data inputs. The app helped clinicians document wound progress more accurately and thoroughly, improving both the quality and consistency of records. This kind of digital support not only enhances clinical decision making but also makes communication across care teams more seamless and transparent.^29^ In a community-based setting, the mobile application PressureUlcerAdvisor app integrated with Braden scale risk assessment tool, educational content, and repositioning reminders designed for informal caregivers was evaluated for its role in supporting preventive risk monitoring. Although the app did not rely on advanced AI, it provided clear guidance and helped caregivers feel more confident in preventing pressure ulcers at home. The tool also promoted consistent monitoring, enabling caregivers to identify and respond to risk factors before complications developed.^30^ Together, these studies highlight the practical value of mHealth tools in wound monitoring. Whether powered by AI or designed for ease of use by nonclinicians, these tools can improve consistency, reduce missed details, and support timely interventions in both clinical and home settings. By enhancing documentation accuracy and supporting continuous tracking, mHealth applications provide a practical way to strengthen the continuity and quality of wound monitoring. These findings are reinforced by feasibility research, as usability studies consistently report that clinicians and caregivers view wound imaging apps as both acceptable and practical for daily use.^16^ At the same time, implementation studies caution that technical barriers such as reliable internet access, device compatibility, and photo quality remain significant obstacles.^18^ Thus, while mHealth tools are valuable for promoting consistency in pressure ulcer monitoring, their ultimate effectiveness depends heavily on contextual factors such as infrastructure readiness and user capacity.

### mHealth in preventing pressure ulcers

Preventing pressure ulcers requires more than clinical knowledge alone, it depends on consistency, vigilance, and the ability to integrate preventive steps like repositioning, risk assessments, and skin checks into daily routines. Yet, in busy or resource-limited care environments, these tasks are often unintentionally missed. Mobile health applications are emerging as helpful companions in this area, offering real-time prompts, education, and support that can turn good intentions into everyday practice. The PressureUlcerAdvisor app provided a combination of educational content, repositioning reminders, and a user-friendly interface tailored to caregivers. As a result, caregivers not only improved their understanding of preventive strategies but also reported feeling more confident and actively engaged in protecting patients from developing ulcers.^30^ Similarly, nurses who used the pressure injury E-book app developed stronger awareness of routine prevention measures, including repositioning techniques and basic skin care.^28^ What stands out in both studies is the shift from passive knowledge to active behavior. In Chuang et al.^28^ the E-book app not only increased nurses knowledge scores but also improved their confidence in applying preventive measures such as repositioning and skin care. Similarly, Polychronis et al.^30^ reported that caregivers using the PressureUlcerAdvisor demonstrated higher self-efficacy in providing preventative care while no significant difference was found in the incidence of new pressure ulcers, the app's reminders and educational content were designed to support adherence to prevention routines.These findings suggest that the apps did more than providing information, they supported users in applying that knowledge in practice, helping to translate awareness into meaningful preventive action. Together, these findings highlight the real-world value of mHealth in reinforcing preventive care. Especially in settings where staffing is stretched or caregivers are not clinically trained, these simple, accessible tools can support habit-building and timely intervention, helping to prevent pressure ulcers before they start. This aligns with broader reviews of electronic clinical decision support systems, which have demonstrated improvements in clinician adherence to preventive guidelines and reductions in healthcare costs, although their effect on pressure ulcer incidence remains inconsistent.^11^ Importantly, feasibility and implementation studies emphasize that prevention apps are most effective when designed with the end user in mind. Older adults and informal caregivers, for example, may encounter digital literacy barriers that restrict meaningful engagement.^19^ Therefore, although the reviewed studies highlight the potential of mHealth to strengthen preventive practices, their real-world adoption will ultimately depend on how well interventions are tailored to user needs and designed to ensure usability across diverse populations.

While the reviewed studies highlighted the potential benefits of mHealth applications, they also pointed out important challenges and limitations. Chuang et al.^28^ noted that although the E-book app improved knowledge, app-based education may not fully replace traditional, hands-on training, and the study was limited to short-term outcomes. Lau et al.^8^ reported variable sensitivity its AI model and only tested its real-world utility using printed wound images, not on actual patients in a clinical setting. Barakat-Johnson et al.^29^ reported that the AI-powered Tissue Analytics app improved documentation and healing outcomes but required reliable image quality, infection control concerns when using phones in patient rooms, and a time-consuming login process. Polychronis et al.^30^ reported that the PressureUlcerAdvisor app improved caregiver confidence and knowledge but also noted that differences in digital literacy among informal caregivers posed a barrier to consistent use. Taken together, these findings indicate that while mHealth applications show considerable promise, their effectiveness is closely shaped by contextual factors such as training support, reliable technical infrastructure, and user readiness.

### mHealth in overall pressure ulcer management

When viewed as a whole, the findings from this review suggest that mHealth applications hold significant promise across the continuum of pressure ulcer care, from early assessment and risk identification to ongoing monitoring and prevention. Their value lies not only in delivering information but also in transforming knowledge into practice. By providing accessible, structured, and often interactive platforms, mHealth tools help translate clinical guidance into everyday routines for both healthcare professionals and informal caregivers. Across the reviewed studies, users reported increased confidence, consistency, and engagement, highlighting the potential of these applications to standardize care, particularly in settings where clinical expertise is limited.

A notable strength of mHealth is its adaptability. These tools were used effectively in hospitals, community settings, and home environments, supporting a range of users from trained nurses to family caregivers. Some applications incorporated advanced features such as AI-driven wound analysis, while others focused on education, reminders, and simple risk assessment tools. Despite their diversity, they shared the common goal of promoting timely, informed, and proactive care, collectively underscoring the potential of mHealth to make pressure ulcer management more responsive, person-centred, and sustainable.

It is also important to recognize that the utility of mHealth applications may vary depending on both the user population and the underlying cause of the pressure ulcer. For example, nurses in acute care may primarily benefit from educational or staging tools, while informal caregivers at home may require simple reminders and structured guidance for daily care. Similarly, pressure ulcers arising from immobility may be more responsive to preventive functions such as repositioning alerts, whereas pressure ulcers linked to neuropathy or sensory loss may require enhanced detection capabilities or targeted caregiver training. Tailoring interventions to these differences will be essential to maximize effectiveness in practice.

These findings should also be considered within the broader digital health landscape. Previous reviews have reported promising advances in wound care applications but have identified challenges related to clinical validation, workflow integration, and user acceptance.^10,13,14^ The successful uptake depends not only on technical functionality but also on adequate training, user trust, and supportive infrastructure.^15^ In line with these observations, the studies included in this review demonstrated encouraging outcomes but were limited by small sample sizes and short follow-up durations, restricting conclusions on long-term effectiveness.^28^ Sustained user engagement also emerged as a challenge, with caregivers sometimes experiencing technology fatigue or inconsistent adherence despite initial acceptance.^30^ Technical barriers including reliable internet connectivity, smartphone literacy, and integration with existing documentation systems were further noted.^8^ Together, these limitations suggest that while mHealth solutions are feasible and beneficial, their routine adoption will require attention to user support, infrastructure, and scalability.

Viewed holistically, the included studies suggest that mHealth applications can strengthen assessment, monitoring, and prevention across diverse contexts. These findings extend earlier feasibility research, which largely emphasized usability and acceptability but rarely demonstrated measurable clinical outcomes.^16,19^ They also resonate with implementation studies, which highlight the importance of workflow integration, sustained engagement, and overcoming barriers such as trust in digital tools and interoperability with health record systems.^18^ By focusing on interventional evidence, this review complements feasibility and implementation work, reinforcing the need for rigorous, long-term evaluations that link innovation with real-world clinical practice.

As digital health continues to evolve, it is essential to design mHealth solutions that are clinically effective, user-centred, accessible, and adaptable across different care settings. Future research should explore integration into broader care ecosystems, supported by training and feedback mechanisms, to ensure sustained impact. mHealth applications hold considerable potential to transform pressure ulcer care, but realizing their full value will require ongoing innovation, robust evaluation, and responsiveness to the needs of both caregivers and patients.

## Limitations

Only a small number of studies met the inclusion criteria, reflecting the early stage of research on mHealth applications in pressure ulcer care. This review focused exclusively on interventional and pilot studies to evaluate the effectiveness of mHealth applications. While this approach allowed us to assess outcome-based evidence, it excluded observational and qualitative studies that may provide important insights into early-stage development, user experience, and real-world adoption of these tools. This may have limited the breadth of perspectives captured in the review. While the findings offer useful insights, they don’t yet allow for broad conclusions about how well these tools work across different settings. The included studies varied in terms of design, sample size, intervention features, and the outcomes they measured, limited the generalizability of findings. Furthermore, the evidence base was not exclusively focused on pressure ulcers. A key study by Barakat-Johnson et al.^29^ included a heterogeneous population with various wound types. Since the positive outcomes were not disaggregated by wound etiology, the generalizability of the quantitative findings specifically to pressure ulcer care is limited. We also focused only on English-language articles that were freely available in full text. This restriction to English-language may have led to the exclusion of relevant studies, introducing potential selection bias which means some relevant work may have been missed. Even so, this review helps lay the groundwork for understanding how mHealth is being used in pressure ulcer care today and points clear directions for future research.

## Conclusion

This scoping review highlights the growing role of mHealth applications in supporting the assessment, monitoring, and prevention of pressure ulcers, particularly where specialist access is limited. These tools ranging from structured educational modules to AI-assisted documentation and caregiver-friendly interfaces offer practical ways to enhance care consistency and user confidence. Although early findings are promising, the current evidence is small, heterogeneous, and largely derived from pilot or quasi-experimental studies. Moreover, some interventions (such as image-based staging classification apps) remain in preliminary validation stages and lack robust accuracy or reliability. Larger, long-term research is needed to evaluate clinical effectiveness, detection accuracy, cost-effectiveness and integration into routine practice across diverse care settings. Future success will depend on designing mHealth tools that are not only clinically effective, but also easy to use, accessible and responsive to real-world caregiving challenges. With continued research and careful implementation, mHealth has strong potential to empower both clinicians and caregivers in delivering better pressure ulcer care.
